# Perceived barriers and facilitators to antiretroviral therapy adherence among youth aged 15–24 years at a regional HIV clinic in SouthWestern Uganda: a qualitative study

**DOI:** 10.4314/ahs.v22i2.7

**Published:** 2022-06

**Authors:** Isaac Jjumba, Micheal Kanyesigye, Gerald Ndagijimana, James Wattira, Clinton Olong, Rose Akumu Olok, Esther Beebwa, Conrad Muzoora

**Affiliations:** 1 Faculty of Medicine, Mbarara University of Science and Technology, Mbarara, Uganda; 2 Department of Nursing, Mbarara University of Science and Technology, Mbarara, Uganda; 3 Department of Internal Medicine, Mbarara University of Science and Technology, Mbarara, Uganda

**Keywords:** Barriers, Facilitators, Adherence, HIV/AIDS, Young Adults

## Abstract

**Introduction:**

South-Western region has the second highest HIV prevalence in Uganda. Youth aged 15–24 have shown poor adherence to antiretroviral therapy compared to the older cohorts. Previous studies from other regions have shown various barriers and facilitators. Our study was designed to describe specific barriers and facilitators to treatment adherence among youths in a large regional HIV clinic in southwestern Uganda.

**Methods:**

We used a phenomenological qualitative study design conducted amongst 30 purposively selected HIV positive youth aged 15–24 years enrolled at Mbarara Regional Referral Hospital HIV clinic on ART for a period of at least one year and 6 key informants using in-depth interviews. The data was collected in an inductive manner during the period between 21st July and 17th August 2020. The recordings were backed up, transcribed verbatim and then analyzed manually using thematic content analysis.

**Results:**

The barriers to ART adherence were described in three descending categories as perceived treatment burden, perceived resultant stigma and discrimination, whereas the main facilitators were; perceived usefulness of HIV medications, availability of free services and Social support

**Conclusion:**

Youths aged 15–24 have challenges with ART associated treatment burden and fear to disclose their HIV status because of the resultant stigma from their communities. Many have however accepted the fact that HIV medications are lifesaving and are strongly motivated to adhere to their medications despite the circumstances.

## Introduction

Human Immunodeficiency Virus (HIV) infection is a life-long chronic infection that causes an acquired Immune deficiency syndrome (AIDS) that has claimed many lives over the last three decades^1^. As per 2018, about 39 million people were living with HIV/AIDS globally with Africa hosting about 22 million, most of whom reside within sub-Saharan Africa^2^. In the same year, a report from Uganda Population-Based HIV impact assessment (UPHIA-2019), estimated 1.4 million Ugandans were living with HIV, and an estimated 23000 Ugandans died of AIDS-related illness^3^. In the same report, the prevalence of HIV among young people in Uganda was 2.1% (3.3% among older adolescent girls and young women and 0.8% among older adolescent boys and young men). It was lower in older adolescents: 1.1% (0.5% for boys and 1.8% for girls) than in young adults: 3.3%, (1.3% for young men and 5.1% for young women).

Although HIV is incurable, Anti-Retroviral Therapy (ART) has proven to be effective in suppressing the virus and prolonging life^4^. However, for ART to work effectively, there is need for optimal adherence of ≥95%^4^. This has been demonstrated to be possible in Africa in the overall adult population although it remains low in specific age groups especially age range of 15–24 where it has been reported to be as low as 26%–29% in a study carried out in Uganda^5^. A study carried out in several countries in the sub-Saharan Africa showed that the same age group experienced substantially higher attrition rates before and after ART initiation compared with their younger adolescents and the older adults^6^. Youths have also been found to have lower rates of retention in care (81%) compared to the those ≥ 25 years (90–94%)^7^.

Several studies mostly performed in urban settings have found out that pill burden, economic constraints, social sexual dynamics, associated drug side effects are main barriers^7^–^10^ whereas psychosocial support, access to free HIV medications, the need to live and fear of opportunistic infections are the main facilitators^11^–^13^. These have however not been studied exhaustively in rural settings^14^. This study was designed to further identify the barriers and facilitators to adherence in a large regional HIV clinic based in a reasonably high HIV prevalent area of South Western Uganda.

## Materials and methods

### Study setting

The study was carried out at Mbarara Regional Referral Hospital (MRRH) HIV Clinic. MRRH is a public non-profit hospital found in Mbarara district, approximately 300km South west of the capital city of Uganda. It provides general out -patient and inpatient care services and has a catchment population of approximately 4 million people coming from mostly rural areas of the entire southwestern region of the country including some from neighboring countries like Tanzania, Rwanda, and Democratic Republic of Congo. The HIV clinic, located within the premises of MRRH, was started in 1998 to provide comprehensive care to HIV infected patients. Cumulatively, it had enrolled approximately 34,000 by July 2020. At that time, there were approximately 11000 patients under active care out of whom approximately 700 were ≤18 years (MRRH HIV Clinic records). Of those in active care approximately 95% were on first line regimens. The Clinic collects data on standardized Ministry of Health (MOH) cards^15^ and maintains a mirror open-MRS electronic database that is updated regularly^16^.

### Study design

We used a phenomenological qualitative study design. Phenomenology is an approach to qualitative research that focuses on the commonality of a lived experience within a particular group. This design was used to describe the specific barriers and facilitators to ART adherence among youth aged 15–24 given that they had similar experiences as far as ART adherence is concerned.

### Study Participants

We included a total of 36 participants (30 youths and 6 health workers). The 30 youths were aged 15–24 years living with HIV/AIDS and accessing ART service at MRRH HIV Clinic for at least one year prior to this study. Of these, 15 (50%) were categorized as having good adherence (≥95%). The six (6) healthcare workers were purposively selected on the basis of their regular interaction with these youths. They consisted counselors (4), clinic nurse (1) and one Clinic medical doctor. This sample size would enable us achieve a saturation point as observed from previous studies^17^.

Study recruitment involved identification of potential participants meeting the study criteria and with scheduled clinic return visits between 21st July 2020 and 08th August 2020. This was done through querying the clinic's electronic records system (OPEN-MRS). We stratified our enrollment by adherence status, age group and sex. Basing on the Ugandan age of consent, we subdivided the participants into <18 years and ≥18. Good adherence in this study was defined as adherence of ≥95% in the preceding 3 clinic visits. The 30 youth participants were purposively sampled keeping the balance of 50% good adherers and 50% less than ideal adherence. For participants aged 18–24 years and key informants we obtained written informed consent directly while for those < 18 years we obtained written informed consent from the guardian/parent and a written informed assent from the participant.

### Study procedures and data collection

The demographics were extracted from the electronic medical record system. The participants were then taken to a private room where in-depth interviews were carried out in a language most understood by the participants which was the Runyankore language using pre-tested interview guides which assessed participant experiences, attitudes, perceptions and knowledge of the various aspects of HIV/ART care like the family, community, health care system, socio-economic aspects, political aspects, academic environment, and personal characteristics (Annex 1). The data was collected by three interviewers. All interviews were audio recorded using SONY ICD-PX470 tape recorder and backed up on Secure Digital (SD) cards for future transcription. We used a similar procedure but a different interview guide (Annex 2) to carry out in-depth interviews with the key informant at their earliest convenience in their private offices. We chose to interview key informants because we believe they are the people who interact most with these youths especially on the most pertinent issues as pertains ART adherence.

### Data management and analysis

During data collection, all the audio recordings were double checked by the principal investigator, copied and backed up daily on external SD cards. Written information about the emotions and physical expressions of the participants were typed and backed up on an external storage device. All audio recordings were transcribed verbatim. Interview scripts were read several times and by the investigators to identify relevant themes in relation to the aim of the study^18^. Data was analyzed manually by thematic content analysis in six phases: familiarization with the data, generating initial codes, searching for sub-categories among the codes, reviewing the merged sub-categories defining and naming the categories^19^.

### Trustworthiness

Credibility was ensured by three of the authors who transcribed and analyzed the data^20^. One of the co-authors, who is a specialist and a researcher in the HIV field, double checked the content /responses of the participants to confirm their relevance^18^. The themes were generated and agreed upon by at least three authors at any time. We stopped at 30 interview scripts because there was no new information that was found out after the initial first 20 scripts because of saturation.

### Ethical considerations

We obtained approval from the HIV clinic, hospital administration and the research ethics committee of Mbarara University of Science and Technology (#13/01-20). Additional approval was obtained from the Uganda National Council for Science and Technology (RESCLEAR/01).

## Results

### Participant characteristics

The study involved a total of 36 participants, 30 youth (16 males and 14 females) (Table 1) and 6 health workers. Thirteen (13) percent of the youth had their highest education level up to primary school, 57% up to ordinary level, 23% up to advanced level and 7% up to tertiary level. We conducted 12 interviews on youth aged 15–17, 18 interviews on youth aged 18–24 and on all the 6 health workers.

**Table 1:** Socio-demographic characteristics

variable	Number
**Sex** Male Female	16 (53%) 14 (47%)
**Age** 15–17 18–24	12 (40%) 18 (60%)
**Education Level** Primary Ordinary Advanced Tertiary	04 (13%) 17 (57%) 07 (23%) 02 (07%)

In this study, perceived treatment burden, perceived stigma, perceived usefulness of medications, health related factors, social support and forgetfulness emerged as the main themes.

### Barriers to ART adherence

#### Treatment burden and drug side effects

Most of the respondents were aware of the usefulness of HIV medications as well as the challenges involved in taking them. Some of these challenges were real life experiences especially the side effects. The commonest reported was dizziness especially when taken on an empty stomach. Fortunately, most knew that taking food, water, juice or porridge was sufficient to prevent this common side effect. Unfortunately, some reported being unable to secure food at the right time of taking the medication either due to food insecurity or other circumstances. Some were students in school where meal times were not flexible where as others were refugees where food was not a guarantee at the desirable time. Almost everyone had a challenge of taking antiretroviral therapy for life, some would prefer simpler formulations like syrup, reduced frequency, smaller tablets or once monthly injections. Two participants expressed this as a barrier:

“*Sometimes the bulk of the drugs scares people, I wish they could make these drugs to be taken like once a week or once a month maybe this would solve the challenge of pill burden*”. B03 male youth, 18 years old.

“*I have a challenge of having to remind myself to take it every day and sometimes when I take it without food it makes me feel weak and this made me to leave it sometimes during periods of scarcity of food until I was counselled*” A05 male youth, 17 years old.

### Stigma, discrimination, and fear of disclosure

Most respondents in this age group would prefer their HIV status to remain anonymous. Most of the respondents were still in school, they expressed a challenge for them to maintain their HIV status undisclosed, especially for those in boarding school where they had to stay with the rest of the students in a confined environment. However, some of them had coped up with the situation by keeping the drugs with people they trust, especially the school nurses. This helped them to keep drugs away from their rooms or with them all the time. For those whose status had been disclosed, they were stigmatized and discriminated against by their colleagues. One participant remarks:

“*The people in the community where I come from talk and this sometimes stigmatizes me and this sometimes makes me hate myself because of their negative comments. One time some village mate of mine happened to come to my school and she spread rumor about my status, this affected, stigmatized me a lot, they used to talk negatively about me and it was worse because I was bright in class so they would say why are you reading? Why are you working hard yet you will die? This all happened while I was in form 3*”. A09 female youth, 17 years old.

### Forgetfulness

A number of our participants reported forgetfulness to take their medications as one of the barriers to ART adherence. They however attributed this to certain distractions like household chores, playing with friends, watching TV and busy schedules for those who work as reported below:

“*Then the other issue is forgetfulness. It's possible for human beings to forget especially when you have many activities with you like you are a child you have to go fetch water, fetch firewood, look after cows, prepare food, wash plates, they are sending you here and there, in that process there is a possibility of one forgetting to take the medicine, they are willing to take but humanly, they forget*”. C05, female counselor.

“*When I had just started I used to forget because I had not gotten used and more so the pressure for work was so much sometimes they could wake me up very early and I leave without taking the drugs*.” B09 male youth, 20 years' old

### Health care related factors

The main challenges that negatively impacted adherence included; frequent drug stock outs which sometimes lead to change of drug regimen, long lines that resulted in prolonged waiting time and occasionally some health workers were perceived as rude and not caring enough. Some of the participants commented as follows;

“*Where I was at first, they could give you courage of going there, whenever we could go there, they could prepare for us porridge, since I reached here [MRRH] I have never been treated in such a way. They could sometimes give us transport and if you have a problem they are there for you. They first teach you before you get your medication. They teach us to feed well, eat greens and things like that, but ever since I reached here [MRRH], I have never attended anything as such. Those lessons they used to teach us would give us courage to adhere to our medications even when you had a feeling of quitting. They would sensitize people. Here [MRRH] you only get taught when you reach the counsellor but we don't see the counsellor each visit*” B05 female youth, 21 years' old

“*Government, sometimes it refuses to pay health workers and you reach the hospital only to find that they are on strike, drug stock outs, one time I came and found that the drugs were few and I was given a refill for only one week and I had to come back after that week*”. A03 male youth, 17 years' old.

### Facilitators to ART adherence

#### No drug, no life; perceived usefulness of HIV medications

The majority of the youths who were interviewed had knowledge that the medication works, they knew that when they take it, their viral load would be suppressed which would enable them to live a longer healthier life and feel better. They clearly understood that without the drugs they would fall sick and probably die. As mentioned by two participants below:

“Since I started taking it [ARVS] I have been in good condition, so I think it can help me preserve my life so that I remain healthy though am HIV positive and I don't have any physical symptoms, B03 male youth, 18 years old.

“*I think the medication is good because when you take the drugs well the virus reduces in the body.........Me I expect that I will keep taking my drugs as I have been advised, I expect that my viral load will be suppressed and I expect that may be in future they will find for us a cure for HIV*”, A08 female youth, 16 years' old

“*I see that it helps, I don't fall sick very often ever since I started taking drugs....... Now government has helped us because without these drugs there is no life*”, A02 male youth, 16 years' old

### Social support

Many respondents acknowledged receiving support from various sources, the most mentioned being family. This was in the form of encouragement, reminders to take medicine, prayers and special attention to their needs. Some received support in the form of counselling from their teachers at school and peers. As highlighted by some two participants below:

“*Family members help so much e.g. sometimes you fall sick of malaria and then you over sleep and you may end up forgetting. But my mother reminds me, in fact she reminds me every day. Then they make sure I have good feeding and they treat me very well. My siblings know that am taking drugs for heart disease that's what my mother told them*”. A08 female youth, 16 years' old “*I have ever stopped taking my medications several times after I heard on radio that taking too many drugs can result into disease. The teachers at school counselled me to take my drugs and I took the advice, then also the gateman where I used to keep the medications also advised me to take my drugs. The counsellors here at the hospital counselled me for like 2 hours and from then I was restored and I started taking the drugs*”. A01male youth,17 years old.

### Health care related factors

Most of the respondents acknowledged that the health care facilities offered useful services in the form of provision of free drugs, counselling and education. Most health care workers were professional and treated the participants well while ensuring confidentiality. One of the participants commented as follows;

“*Then health workers, they are easy to me, I always come when am rushing and they work on me very fast because they know me and I always come early.........Yeah the government supports us because it provides us with free drugs and that's the main issue to me.........First of all, counselling is the whole thing, the counselling package from an experienced counsellor is enough to bring someone back on track*” A08 female youth, 16 years old.

## Discussion

This was a study carried out on youths aged 15–24 years to assess for perceived barriers and facilitators to ART adherence in a large regional HIV clinic in southwestern Uganda.

We found that most participants faced challenges which included; treatment burden, drug side effects, stigma, discrimination, fear of disclosure, forgetfulness to take medications and health care system. On the other hand, the main facilitators included; perceived usefulness of ART medications, social support, and health care related factors.

Many respondents had problems with ART medications expressed as the fact that they are life long and are associated with side effects. This is not new and has been shown from previous studies^21^–^24^. Other drug related challenges included pill burden (Expressed in frequency of taking the pills, pill size and formulation) as was shown in a systematic review across sub-Saharan Africa^23^. The requirement to take drugs with food and balanced diet raised the obvious issue of food insecurity in sub Sahara Africa and is not new in youths living in low income countries 25. This has been found elsewhere, most especially for youths raised by single parents, in refugee camps and in war torn areas^26^–^28^.

In this study, we also found out that most youth preferred to keep their HIV status anonymous. This is not a unique finding and has been reported by several studies^29^–^32^. The majority of our participants were in boarding schools and expressed fear of being treated differently/discriminated against in the event of disclosure of HIV status. This appears to be a common reason for non-disclosure as has been described elsewhere^33^–^37^. In some cases, where the HIV status was disclosed, some experienced stigma and discrimination in the form of rejection, discouragemnt and isolation. Such experiences have been faced by youths elsewhere^34^, ^36^.

Forgetfulness to take their medications featured as one of the barriers to ART adherence. Studies have reported several reasons like sleeping away from home^38^, house chores^39^, watching TV^40^. A randomized control trial about use of mobile technologies in a resource limited setting found out that mobile phone reminders were effective means of improving adherence^41^. We found out that in youth who work, busy schedules and distractions were the major cause of forgetfulness. This was also found by Nabukeera et al 2015 in Uganda^15^, ^41^.

The respondents perceived the health care system as both an enabler and a barrier at the same time. Most youths appreciated provision of free drugs and free HIV services. They also appreciated the counselling and support from the health workers. These findings have been re-echoed in other studies across sub-Saharan Africa^42^–^45^. However, some complained about the long lines at the clinics leading to long waiting times as is the case in similar resource limited settings elsewhere^46^. In some studies, patients have been forced to change regimen because of drug stock outs and sometimes this lead to drug resistance^29^, ^47^

The most important facilitator in this study was the perceived usefulness of the HIV medication. Almost everyone knew that these medicines were very useful in improving and prolonging life (No drug no life). Most even knew the mechanism by which the drugs work; decreasing the viral load and improving their immunity. This has also been the case in a related study carried out in Botswana^48^ often referred to as self-efficacy of the medications^49^. Another enabler worthy to note is thatome respondents took the drugs to prevent showing signs of HIV/AIDS disease. They wanted to look healthy and normal like others. This is similar to what was found in a study carried out in Malawi^21^.

In general, many respondents perceived social support as a very important enabler for good adherence as it has been found in several other similar studies^47^,^50^–^52^. We found that families were most important providers of psychosocial support in the form of special care like ensuring timely availability of food to facilitate medications, family members also play a very important role in reminding these youths to take their medications at the right time, encouragement and counselling and family support has also been demonstrated to be important from a study in southern Uganda^51^. Financial support is also another form of assistance that family offers to these youths. Some respondents received similar support from teachers, nurses and matronst school as has been found elsewhere in similar studies^51^, ^53^.

In conclusion, our study findings confirm most of what has been found elsewhere, especially in sub-Sahara Africa. Youths, especially those in school face major challenges to ART adherence the most important being stigma and discrimination. It is the single most important factor that hinders disclosure of their HIV status due to the fear of being treated differently.

## Limitations

The main limitation of our study was the emergency of COVID-19 at the start of our enrollment. This triggered nationwide lockdowns that severely hindered transportation, a number of our sampled participants could not make it for the appointment, we were therefore forced to sample from only those who could make it to the clinic, however, the ability to make it to the clinic in such circumstances is also strongly correlated with health seeking behavior, socio-economic status, and proximity to the HIV clinic all the above factors are strongly associated with good adherence. There is therefore a possibility that we did not get enough representation of participants with long-term poor adherence (Beyond the 3 recent visits).

## Recommendations

We recommend strategies be put in place, especially by boarding schools to support these youths and also students at school should be sensitized about the effects of stigma and discrimination to people living with HIV/AIDS.
